# Population pharmacokinetics of unbound cefazolin in infected hospitalized patients requiring intermittent high-flux haemodialysis: can a three-times-weekly post-dialysis dosing regimen provide optimal treatment?

**DOI:** 10.1093/jac/dkae318

**Published:** 2024-09-10

**Authors:** Carleigh Duke, Suzanne L Parker, Betty B Zam, Fabian Chiong, Cherian Sajiv, Basant Pawar, Aadith Ashok, Brynley P Cooper, Steven Y C Tong, Sonja Janson, Steven C Wallis, Jason A Roberts, Danny Tsai

**Affiliations:** College of Medicine and Public Health, Flinders University, Corner Skinner and Simpson Streets, Darwin 0870, Northern Territory, Australia; University of Queensland Centre for Clinical Research, University of Queensland, Brisbane, Queensland, Australia; Pharmacy Department, Alice Springs Hospital, Alice Springs, Northern Territory, Australia; Department of Medicine, Alice Springs Hospital, Alice Springs, Northern Territory, Australia; Department of Nephrology, Alice Springs Hospital, Alice Springs, Northern Territory, Australia; Victorian Infectious Diseases Service, The Royal Melbourne Hospital at the Peter Doherty Institute for Infection and Immunity, Melbourne, Australia; Department of Medicine, Alice Springs Hospital, Alice Springs, Northern Territory, Australia; Pharmacy Department, Alice Springs Hospital, Alice Springs, Northern Territory, Australia; Department of Nephrology, Alice Springs Hospital, Alice Springs, Northern Territory, Australia; Department of Infectious Diseases, The University of Melbourne at the Peter Doherty Institute for Infection and Immunity, Melbourne, Australia; Department of Infectious Diseases, Royal Darwin Hospital, Darwin, Northern Territory, Australia; University of Queensland Centre for Clinical Research, University of Queensland, Brisbane, Queensland, Australia; University of Queensland Centre for Clinical Research, University of Queensland, Brisbane, Queensland, Australia; Department of Intensive Care Medicine, Royal Brisbane and Women’s Hospital, Brisbane, QLD, Australia; Herston Infectious Diseases Institute (HeIDI), Metro North Health, Brisbane, QLD, Australia; Division of Anaesthesiology Critical Care Emergency and Pain Medicine, Nîmes University Hospital, University of Montpellier, Nîmes, France; College of Medicine and Public Health, Flinders University, Corner Skinner and Simpson Streets, Darwin 0870, Northern Territory, Australia; University of Queensland Centre for Clinical Research, University of Queensland, Brisbane, Queensland, Australia; Pharmacy Department, Alice Springs Hospital, Alice Springs, Northern Territory, Australia

## Abstract

**Objectives:**

To describe the population pharmacokinetics of cefazolin in infected hospitalized patients requiring intermittent haemodialysis (IHD).

**Methods:**

This prospective population pharmacokinetic study was conducted in IHD patients prescribed cefazolin 2 g three times weekly. Plasma samples were collected at prespecified timepoints and assayed for total and unbound concentrations using validated LC. Pharmacokinetic modelling and dosing simulations were performed using Pmetrics^®^. PTA in plasma suitable for MSSA (unbound trough concentrations of ≥2 mg/L for the final 24 h of a 72 h interval) were simulated for different dosing regimens. A PTA of ≥95% was deemed acceptable.

**Results:**

A total of 260 cefazolin concentrations (130 total, 130 unbound) were collected from 16 patients (14 female) with a median age of 51 years. The median (IQR) pre-dialysis unbound cefazolin concentration for a 3 day dose interval trough was 17.7 (13.5–31.4) mg/L. The median (IQR) unbound fraction was 0.38 (0.32–0.46). The lowest pre-dialysis unbound concentration was 9.1 mg/L. A two-compartment model with a complex protein-binding component adequately described the data. The mean unbound cefazolin CL during IHD was 16.4 ± 4.26 L/h, compared with 0.40 ± 0.19 L/h when dialysis was off. Duration of time on haemodialysis (TOH) was the only covariate supported in the final model. The 2 g three-times-weekly regimen was associated with a PTA of 99.7% on dosing simulations to maintain unbound concentrations of ≥2 mg/L with TOH of 6 months. The 1 g three-times-weekly post-dialysis was associated with a PTA of 95.4%.

**Conclusions:**

A 2 g three-times-weekly post-dialysis cefazolin regimen is supported for MSSA infections.

## Introduction

Indigenous populations across the globe are known to experience an increased incidence of chronic kidney disease.^1^ The prevalence of Indigenous Australians with end-stage kidney disease (ESKD) and requiring intermittent haemodialysis (IHD) is reported to be 6–15 times higher than non-Indigenous Australians.^2–4^ Indigenous Australians requiring IHD have an incidence of infection-associated hospitalization up to 11.9 per 100 patient years.^5^ Furthermore, IHD has been significantly associated with *Staphylococcus aureus* bloodstream infection due to venous access required for dialysis, particularly indwelling catheters.^6–8^ Infection in ESKD patients requiring renal replacement therapy is a leading contributor to morbidity, mortality and hospitalization worldwide, accounting for 12%–36% of mortality in this patient population.^9–11^ The reported incidence of infection-associated deaths in individuals with ESKD is 1.7/100 patient years (95% CI 1.47–1.86) in Australia and New Zealand, with an increased risk for Indigenous Australians.^12^

Cefazolin is a first-generation cephalosporin frequently used as an empirical agent for skin-and-soft-tissue infections in patients on IHD.^13^ It exhibits bactericidal properties against Gram-positive pathogens including MSSA and *Streptococcus pyogenes*.^13^ Cefazolin’s pharmacodynamics are time-dependent in nature and its efficacy is determined by *fT*_>MIC_.^14^ Cefazolin is widely distributed to soft tissues, highly protein bound in healthy individuals (80%–90%) and primarily cleared via the renal route (80%–100% of dose is recovered in urine).^13,15^ In ESKD, cefazolin half-life can extend beyond 20 h (non-dialysis) and a three-times-weekly post-dialysis cefazolin dosing strategy (through existing vascular access for dialysis) has been previously proposed for patients requiring IHD.^13^ When compared with conventional daily regimens, this dosing method improves convenience and minimizes venepuncture and insertion of peripheral venous access devices, thereby preserving the longevity of veins likely required for future vascular access.^16^ However, most pharmacokinetic studies of cefazolin administered post-dialysis have only measured total cefazolin concentrations (unbound concentrations not tested), used inconsistent MIC targets, and frequently included patients without infection.^13,17–19^ As such, a consensus has not been reached on an optimal dose to be recommended for the 72 h interdialytic interval of IHD (2 g, 3 g or weight-based) for a three-times-weekly post-dialysis regimen.

Based on the pharmacokinetic properties of cefazolin, it is unlikely to produce interethnic differences.^20^ Nonetheless, there are relevant chronic physiological changes associated with ESKD and IHD, and the high incidence of bloodstream infection and skin-and-soft-tissue infections reported in Indigenous Australians.^21,22^ We aimed to evaluate the pharmacokinetics of cefazolin, in both total and unbound concentrations, in Indigenous Australians requiring IHD, and describe the PTA of a 2 g three-times-weekly post-dialysis regimen.

## Materials and methods

### Setting and study population

This was a single-centre, prospective population pharmacokinetic study conducted in the renal dialysis unit of a remote Australian hospital. Participants were included in the study if they met the following criteria: (i) Australian Indigenous; (ii) ≥18 years of age; (iii) on three-times-weekly high-flux haemodialysis; and (iv) prescribed with a cefazolin 2 g three-times-weekly post-dialysis regimen. Participants were excluded if they were: (i) pregnant; (ii) had a documented allergy to cefazolin or other cephalosporins; or (iii) required more frequent dialysis. Ethical approval was obtained from both local and university Ethics Committees (Central Australian Human Research Committee, project code CA-19-3345, and The University of Queensland Human Research Ethics Committee, project code 2019/HE002332). Study participants provided written consent before enrolment.

### Dosing regimen and blood sampling

The regimen of three-times-weekly cefazolin 2 g post-dialysis dosing is typically used at the study site. Each 2 g dose of cefazolin (Cefazolin-AFT^TM^; AFT Pharmaceuticals, NSW, Australia) was made up with 10 mL of ‘water for injection’ and injected through the patient’s arterial venous fistula or their central line as a slow push over 5 min at the completion of the corresponding dialysis session. Blood was sampled in 0.5 mL aliquots and collected in lithium-heparin tubes over two dosing or dialysis intervals at the following times: directly prior to dialysis, immediately after dialysis, then at 5, 15, 60 and 1440 min following the dose of cefazolin, then again at 48 h or immediately prior to the next dialysis session (whichever came first), and prior to the next dialysis session when the dialysis interval was 72 h. Times for each patient varied slightly depending on clinical management. Exact timing of sampling was recorded. The dialysers utilized at the centre were: FX80, FX100 and FX120 (Fresenius Medical Care, Hesse, Germany), with ultrafiltration coefficients of 59, 73 and 87 mL/h/mmHg, and surface areas of 1.8, 2.2 and 2.5 m^2^, respectively. This sampling regimen has been described in previous papers.^23^ Demographics and clinical data were collected from patient medical records and communication with participants. Clinical data collected include indication for cefazolin therapy and adverse drug reaction after the administration of cefazolin.

### Storage and handling of samples

Once collected, samples were immediately stored at 2°C–8°C. Within 4 h of collection samples were centrifuged at 5000 rpm for 6 min at 2°C–8°C. The plasma was then aspirated into cryovials and stored at −80°C until transfer to the University of Queensland Centre for Clinical Research bioanalytical laboratory for drug assay.

### Drug assay

The plasma total and unbound concentrations of cefazolin were measured by a validated UHPLC-MS/MS; see Table S1 (available as Supplementary data at *JAC* Online) for the detail of the assay. The unbound fraction was isolated by ultrafiltration at 37°C with Centrifree devices (Merck Millipore, Tullagreen, Ireland). Test samples were assayed in batches alongside calibrators and quality controls and results were subject to batch acceptance criteria.^24^

### Population pharmacokinetic analysis

One- and two-compartment pharmacokinetic models were developed using the Pmetrics^®^ software package (version 1.9.7)^25^ for R^®^ (version 4.1.2). The models were constructed with the non-parametric adaptive grid (NPAG) algorithm using the unbound and total plasma cefazolin concentrations and timepoint data, dosing administration timepoints, time and duration of IHD sessions. Dialysis was included in the model using selective execution of a logical expression statement (see Table S2). Demographic data [weight, age, sex, albumin concentrations, liver transaminase concentrations, duration of time the patient had been commenced on IHD therapy for ESKD treatment (time on haemodialysis; TOH) and type of dialyser filter used] were tested for inclusion in the model as covariates.

Due to the high albumin-binding characteristic of cefazolin, numerous binding models were tested for one that best describes the concentration–time data collected.^26^ We initially tested simple binding models, where a linear relationship was expected between unbound and total cefazolin concentrations:


$$C_{\mathrm{unbound}}=C_{\mathrm{total}\;}\times \;\mathrm{FF}\;\times \;\frac{39}{\mathrm{Alb}}$$

where C_unbound_ and C_total_ are the unbound and total cefazolin concentrations in mg/L, respectively, FF is the unbound (free) fraction of cefazolin, and Alb is the serum albumin concentration in g/L.

A previously published complex binding model designed to describe the pharmacokinetics of high protein-binding drugs was also tested.^26,27^


$$C_{\mathrm{unbound}}=C_{\mathrm{total}}-\;\frac{B_{\max}\;\times \;C_{\mathrm{unbound}}}{K_{D}+\;C_{\mathrm{unbound}}}$$

$$B_{\max}\;=\mathrm{Alb}\;\times \;N\;\times \;\frac{M_{\mathrm{CFZ}}}{M_{\mathrm{Alb}}}\;\times \;1000$$

$$K_{D}=\;\frac{1}{K_{A}}=\;\frac{K_{\mathrm{off}}}{K_{\mathrm{on}}}$$

where B_max_ is the maximum cefazolin binding concentration (mg/L), K_D_ is the equilibrium dissociation constant for cefazolin–albumin (mg/L), N is the number of cefazolin binding sites on each albumin molecule, M_CFZ_ is the molecular weight of cefazolin (g/mol), M_Alb_ is the molecular weight of albumin (g/mol), K_A_ is the equilibrium association constant (L/mg), K_off_ is the first-order cefazolin-binding dissociation rate constant (h^−1^), and K_on_ is the second-order cefazolin-binding association rate constant (L/mg/h). Cefazolin is reported, on average, with 0.6 binding sites for each albumin molecule.^28^ The molecular weights of cefazolin and albumin are 455 and 66 500 g/mol, respectively. The final pharmacokinetic model is presented in Table S3, where we have included B_max_, K_off_ and K_on_ in the model.

For the development of the cefazolin pharmacokinetic model, a statistically significant improvement between two nested models was achieved when a reduction in the Akaike information criterion (AIC) and Bayesian information criterion (BIC), and a decrease of >3.84 in log-likelihood ratio (2*LL) for both the inclusion of a model parameter or a covariate was required. Backward elimination of model covariates was supported when elimination resulted in an increase of >6.64 in the AIC, BIC and 2*LL was required.

For covariate assessment, physiological variables that can plausibly affect cefazolin’s pharmacokinetic parameters [central volume of distribution (*V*_c_), unbound cefazolin CL, K_on_, and K_off_] were assessed for statistical relationship and tested for inclusion into the model following the parameter acceptance sequence described above.

### Model diagnostics

Evaluation of the final pharmacokinetic model was conducted by visually assessing the population and individual goodness-of-fit plots of observed and predicted concentrations of cefazolin. The predictive performance was also assessed for mean prediction error and the mean bias-adjusted squared of the population and individual posterior predictions. A predictive plot was visually assessed to determine the model validity.

### Monte Carlo dosing simulation

Monte Carlo dosing simulations were performed using Pmetrics in R^®^ (*n* = 1000), and PTA was simulated for unbound cefazolin concentrations achieved in plasma against various dosing strategies.

The overall pharmacokinetic/pharmacodynamic target was maintaining unbound plasma cefazolin concentration above 2 mg/L for 100% of the final 24 h of a 72 h dose interval (100%*fT*_>MIC 48–72_). MIC_50_ and MIC_90_ have been reported as 0.5 and 1 mg/L for MSSA infections, respectively.^29^ Furthermore, the majority of MSSA infections have an MIC of ≤2 mg/L reported by EUCAST,^30^ for which cefazolin is most commonly indicated.

The dosing regimens that were tested in the simulations were described in a similar study,^23^ assuming IHD occurs on Days 1, 4, and 6: 1 and 2 g post-dialysis three times weekly; 3 g post-dialysis on Day 1 (prior to a 3 day interdialytic period); and 2 g post-dialysis on Days 4 and 6 (prior to 2 day interdialytic periods); and 0.5 and 1 g daily (post-dialysis on dialysis days), across the number of months since initiation of IHD (TOH) of 6, 12, 24, 36 and 60 months, for the MICs of 0.125, 0.25, 0.5, 1, 2, 4, 8 and 16 mg/L. A higher than conventionally accepted target PTA of ≥95% was selected due to the greater impact of infections on morbidity and mortality in this high-risk population.^23^

## Results

### Study population

A total of 130 samples with paired total and unbound cefazolin plasma concentrations for 16 patients were available for pharmacokinetic analysis. The demographics and clinically relevant information are detailed in Table 1. Plasma cefazolin concentrations are presented in Table 2. Overall, the cohort were largely female (88%) and the median age (IQR) of the population was 51 (38.8–62.3) years. No adverse drug reactions were reported. The median (IQR) unbound fraction was 0.38 (0.32–0.46) and the median (IQR) pre-dialysis unbound cefazolin concentrations of a 2 day and 3 day dose interval were 35.7 (27.5–45.7) and 17.7 (13.5–31.4) mg/L, respectively. The lowest pre-dialysis unbound concentration was 9.1 mg/L. Dialysis parameters are presented in Table S4.

**Table 1.** Patient demographics, clinical information and concentration–time point data

Parameter	*n* = 16
Age, years, median (IQR)	51 (38.8–62.3)
Female, *n* (%)	14 (88)
Weight, kg, median (IQR)	69.5 (58.5–76.3)
TOH, months, median (IQR)	59 (24.3–120)
Indication for cefazolin therapy	Line-associated cellulitis (4) Wound infection (3) Abscess (3) Diabetic foot infection (2) Periorbital cellulitis (2) Bacteraemia (3) Surgical prophylaxis (1)
Serum albumin, g/L, median (IQR)	38.5 (35.5–40)
Serum bilirubin, µmol/L, median (IQR)	7.5 (6–12)
ALP, U/L, median (IQR)	233 (179–307)
GGT, U/L, median (IQR)	142 (71–192)
ALT, U/L, median (IQR)	9 (5.75–18.75)
Pre-dialysis urea, mmol/L, median (IQR)	19.6 (16.2–22.8)
Filter used, *n* (%)	FX80–5 FX100–10 FX120–1

ALP, serum alkaline phosphatase concentration.

**Table 2.** Plasma cefazolin concentration data

Parameter	*n* = 16
Unbound fraction, median (IQR)	0.38 (0.32–0.46)
Dialysis duration, h, median (IQR)	4.0 (4.04–4.27)
Pre-dialysis total cefazolin troughs at 48 h, mg/L, median (IQR)	98.7 (76.6–114.3)
Pre-dialysis unbound cefazolin troughs at 48 h, mg/L, median (IQR)	35.7 (27.5–45.7)
Pre-dialysis total cefazolin troughs at 72 h, mg/L, median (IQR)	53.0 (38.2–67.2)
Pre-dialysis unbound cefazolin troughs at 72 h, mg/L, median (IQR)	17.7 (13.5–31.4)
Total cefazolin concentration reduction during each dialysis session, %, median (IQR)	72.6 (69.2–75.8)
Unbound cefazolin concentration reduction during each dialysis session, %, median (IQR)	83.3 (78.7–86.3)

### Population pharmacokinetic model and model diagnostics

A two-compartment model incorporating a complex protein-binding component was found to best describe the data collected. The pharmacokinetic parameter estimates are presented in Table 3. The mean unbound cefazolin CL when dialysis was off versus on was 0.40 ± 0.19 versus 16.36 ± 4.26 L/h, respectively. The duration of time the patient had been commenced on IHD therapy as part of their ESKD treatment (TOH) demonstrated an inverse-power relationship with unbound cefazolin CL (r^2 ^= 0.433) and was the only covariate retained in the final pharmacokinetic model. The final model file and a diagram depicting the compartments are presented in Table S3 and Figure S1, respectively).

**Table 3.** Cefazolin pharmacokinetic model parameter estimates

Parameter	Mean	SD	CV (%)	Median	Shrinkage (%)
CL_nHD_ (L/h)	0.40	0.19	46.00	0.39	0.09
CL_HD_ (L/h)	16.36	4.26	26.04	16.31	0.30
*V* (L)	25.02	7.55	30.16	25.24	—^a^
*V*_c_ (L)	6.51	1.30	20.02	6.74	0.27
t_½nHD_ (h)	65.31	63.21	96.79	41.12	—^a^
t_½HD_ (h)	1.08	0.27	24.48	1.16	—^a^
K_on_ (L/mg/h)	2.17	0.56	25.86	2.39	0.17
K_off_ (h^−1^)	92.13	15.15	16.44	89.83	0.67
K_cp_ (h^−1^)	4.01	2.54	63.23	3.51	0.15
K_pc_ (h^−1^)	1.72	1.48	86.22	1.17	0.19

t_½nHD,_ t_½_ when dialysis is off; t_½HD,_ t_½_when dialysis is on; K_cp_, rate transfer constant from the central to peripheral compartment; K_pc_, rate transfer constant from the peripheral to central compartment; CV, coefficient of variation.

^a^Data not available as the entry is manually calculated.

The final CL model is as follows.

When dialysis is off:


$$\mathrm{CL}=CL_{\mathrm{nHD}}\;\times \;{\left(\;\frac{59}{\mathrm{TOH}}\right)}^{0.28}$$

When dialysis is on:


$$\mathrm{CL}=CL_{\mathrm{HD}}$$

where CL is the population parameter estimate of unbound cefazolin clearance, in L/h, CL_nHD_ is the population parameter estimate of unbound cefazolin CL when dialysis is off, in L/h, TOH is the time since initiation of haemodialysis in months, CL_HD_ is the population parameter estimate of unbound cefazolin CL when dialysis is on, in L/h.

During the process of model design and covariate inclusion, the quantitative demographic, clinical and concentration–time data were incorporated into the following models: one-compartment model with simple binding, one-compartment model with a complex protein-binding component, two-compartment model with simple binding, two-compartment with a complex binding component, and two-compartment with a complex binding component in addition to the covariate of TOH. The comparison of AIC, BIC and 2*LL between these pharmacokinetic models is presented in Table S2.

**Figure 1.**
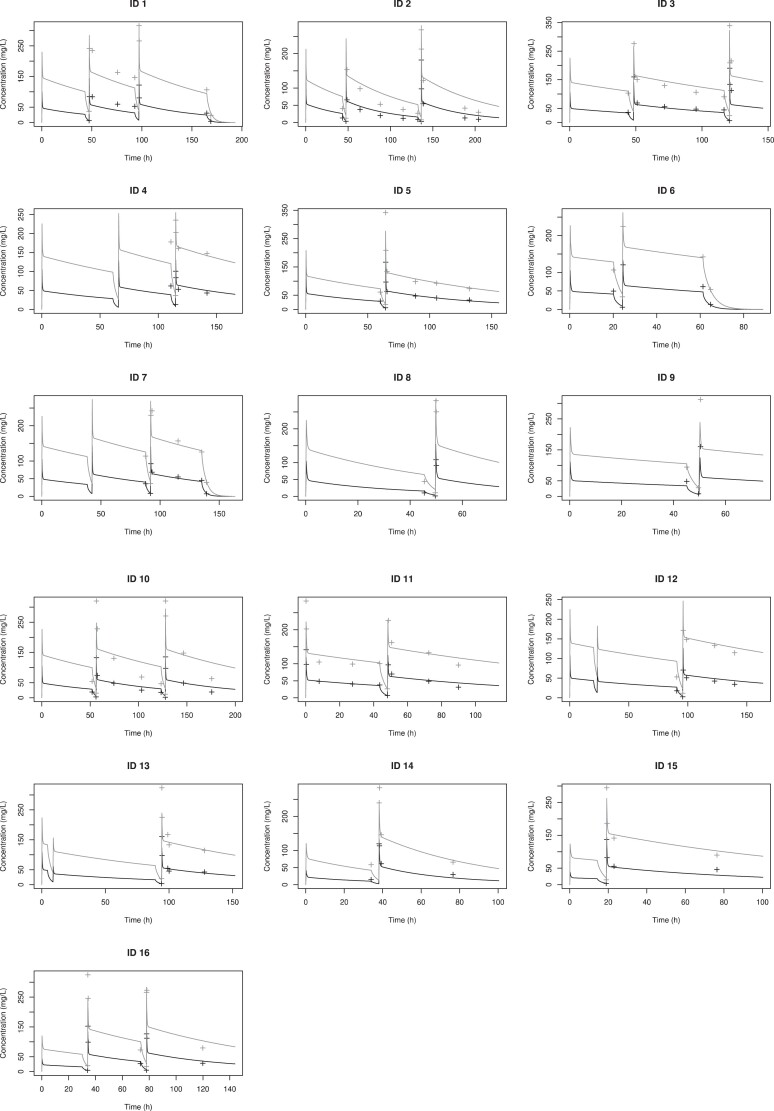
Observed total (grey cross) and unbound (black cross) cefazolin concentration–time data are presented with predicted lines of the final model (total, grey line; unbound, black line) for each individual patient.

**Figure 2.**
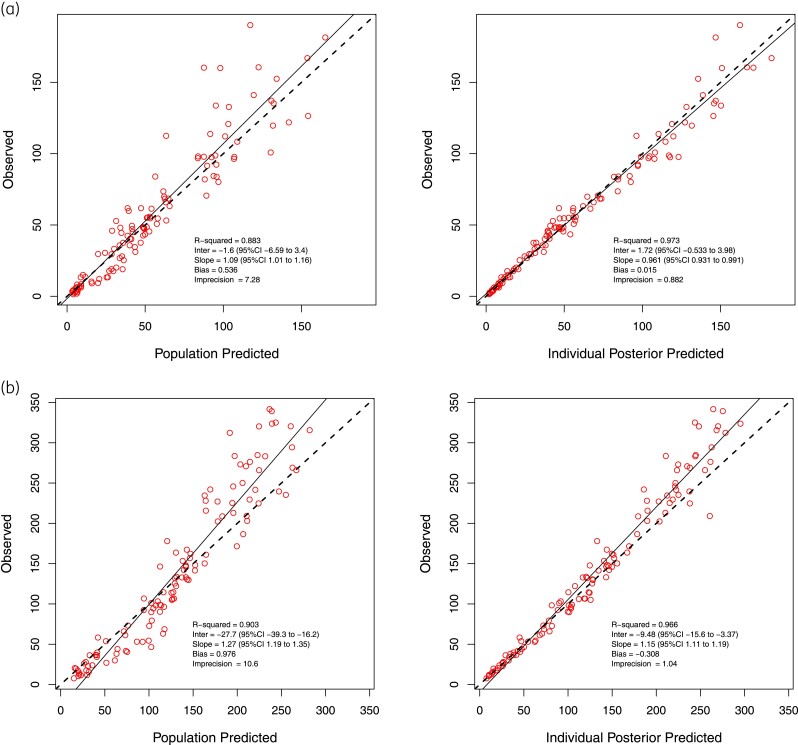
Observed-versus-predicted goodness-of-fit plots for unbound and total cefazolin concentration (open circles). (a) Population- and individual-predicted unbound cefazolin concentration; (b) population- and individual-predicted total cefazolin concentration. The cefazolin concentration shown are in mg/L. This figure appears in colour in the online version of *JAC* and in black and white in the print version of *JAC*.

**Figure 3.**
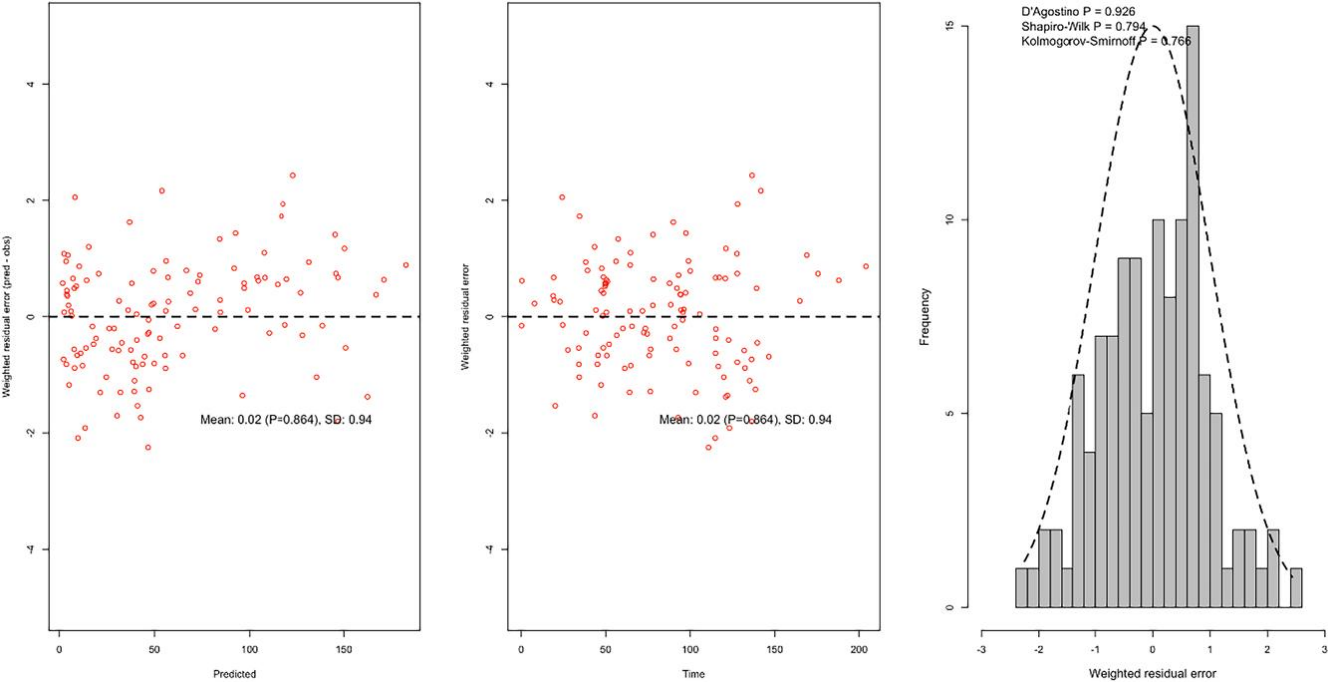
Residual diagnostic plots for unbound cefazolin concentrations. From left to right: weighted residual error versus predicted concentration; weighted residual error versus time; weighted residual error frequency histogram. This figure appears in colour in the online version of *JAC* and in black and white in the print version of *JAC*.

The diagnostic plots of observed cefazolin concentration–time data (total and unbound) against the final model for each individual patient presented in Figure 1 were deemed acceptable when examined visually. The observed-versus-predicted goodness-of-fit plots for unbound and total cefazolin concentrations, and the residual diagnostic plots are presented in Figures 2 and 3.

### Dosing simulations

A cefazolin 2 g post-dialysis dose with a 72 h dose interval was associated with a PTA of 99.7% for patients with TOH of 6 months. The PTA of various dosing regimens against different TOHs are presented in Table 4. All tested regimens resulted in a PTA of >95%.

**Table 4.** PTA of 100% *fT*_>MIC_ (%) on the last day of a 3 day dose interval for various dosing regimens

Dosing regimen	TOH (months)	PTA of 100% *fT*_>MIC_ (%) for MIC (mg/L) of:							
		0.125	0.25	0.5	1	2	4	8	16
1 g, 1 g, 1 g	6	**100**	**100**	**100**	**100**	**95.4**	62.3	29.5	6
	12	**100**	**100**	**100**	**100**	**99.9**	84.5	40.3	1.5
	24	**100**	**100**	**100**	**100**	**100**	**98**	50.3	5
	36	**100**	**100**	**100**	**100**	**100**	**99.9**	58.2	9.2
	60	**100**	**100**	**100**	**100**	**100**	**100**	75.6	15.1
2 g, 2 g, 2 g	6	**100**	**100**	**100**	**100**	**99.7**	90.6	56.6	27.9
	12	**100**	**100**	**100**	**100**	**100**	**99**	77.6	39.5
	24	**100**	**100**	**100**	**100**	**100**	**100**	94.8	48.9
	36	**100**	**100**	**100**	**100**	**100**	**100**	**98**.**8**	54.6
	60	**100**	**100**	**100**	**100**	**100**	**100**	**100**	68.6
3 g, 2 g, 2 g	6	**100**	**100**	**100**	**100**	**100**	**96**	74.4	44.4
	12	**100**	**100**	**100**	**100**	**100**	**99.9**	91.3	54.2
	24	**100**	**100**	**100**	**100**	**100**	**100**	**99**.**2**	71.8
	36	**100**	**100**	**100**	**100**	**100**	**100**	**100**	84.9
	60	**100**	**100**	**100**	**100**	**100**	**100**	**100**	**96**.**8**
500 mg q24h	6	**100**	**100**	**100**	**100**	**100**	**100**	**98**.**2**	40.4
	12	**100**	**100**	**100**	**100**	**100**	**100**	**99**.**8**	54
	24	**100**	**100**	**100**	**100**	**100**	**100**	**99**.**9**	72.2
	36	**100**	**100**	**100**	**100**	**100**	**100**	**100**	83.4
	60	**100**	**100**	**100**	**100**	**100**	**100**	**100**	**94**.**8**
1 g q24h	6	**100**	**100**	**100**	**100**	**100**	**100**	**100**	**95**.**5**
	12	**100**	**100**	**100**	**100**	**100**	**100**	**100**	**99**.**7**
	24	**100**	**100**	**100**	**100**	**100**	**100**	**100**	**99**.**9**
	36	**100**	**100**	**100**	**100**	**100**	**100**	**100**	**100**
	60	**100**	**100**	**100**	**100**	**100**	**100**	**100**	**100**

1 g, 1 g, 1 g, a dosing regimen of 1 g on Day 1, 1 g on Day 4 and 1 g on Day 6; 2 g, 2 g, 2 g, a dosing regimen of 2 g on Day 1, 2 g on Day 4 and 2 g on Day 6 of a 7 day course; 3 g, 2 g, 2 g, a dosing regimen of 3 g on Day 1, 2 g, on Day 4 and 2 g on Day 6 of a 7 day course; 500 mg q24h, a dosing regimen of 500 mg once daily; 1 g q24h, a dosing regimen of 1 g once daily. The bold values are >95%.

## Discussion

Our findings support the use of a 2 g three-times-weekly post-dialysis dosing regimen for the treatment of infections with bacteria for which the cefazolin MIC is ≤2 mg/L, regardless of when the patient commenced IHD therapy for ESKD.

The fixed dosing schedule of 2 g cefazolin three times weekly post-dialysis had been previously described by Kuypers *et al.* and Fogel *et al.*^18,31^ Kuypers reported mean pre-dialysis trough concentrations of 84, 97 and 61 mg/L on Days 2, 4 and 7, respectively, following a 2 day, 2 day and 3 day dose-interval regimen.^31^ In our study, we report a similar finding of median total pre-dialysis trough concentrations of 98.7 (76.6–114.3) and 53.0 (38.2–67.2) mg/L following a 2 day and 3 day dose interval, respectively. We further report median (IQR) unbound trough concentrations of 35.7 (27.5–45.7) and 17.7 (13.5–31.4) mg/L for these respective dosing intervals, where no patients had unbound trough concentrations below 2 mg/L throughout all interdialytic intervals. The 99.7% PTA for this regimen further supports its use in the clinical setting.

Our study reports a mean unbound cefazolin CL of 0.40 ± 0.19 L/h, which is slightly higher than for other ESKD populations (approximately 0.20 L/h), potentially due to their focus on systemic clearance of total cefazolin concentrations rather than unbound concentrations, as well as a difference in residual renal function.^32^ In our study, we noted an inverse correlation between cefazolin clearance and TOH, which can be seen as a surrogate for the incremental reduction in the residual renal function from the initiation of haemodialysis therapy, which, to the best of our knowledge, has not been previously included in pharmacokinetic models for patients requiring IHD. This finding is likely to be clinically relevant when devising the dosing of antimicrobials that are predominantly cleared via the renal route, whereby a small change in the renal clearance can induce significant changes in plasma concentrations. By including this covariate in the final pharmacokinetic model, the PTA (100%*fT*_>MIC_ during the last 24 h of a 72 h interval) of the 2 g three-times-weekly regimen increased from 99.7% to 100% when TOH is increased from 6 to 12 months. Nonetheless, the lowest pre-dialysis trough concentration we have observed is 9.1 mg/L, which is well over the 2 mg/L target MIC.

We observed the total and unbound concentrations of cefazolin to reduce by 72.6% and 83.3%, respectively, during the dialysis session. This is compared with 60% and 62% reduction in total cefazolin concentrations reported by Marx *et al.* and Sowinski *et al.*, respectively, who both used high-flux dialysis.^19,32^ The high-flux membranes used in our study (FX80, FX 100 and FX120; Fresenius, Lexington, MA, USA) have ultrafiltration coefficients of 64, 74 and 87 mL/h/mmHg, respectively, while membranes used by Sowinski *et al*. and Marx *et al*. have coefficients between 36 and 55 mL/h/mmHg.^19,32^ We did not find a significant correlation between CL and the different membranes, which may be due to the relatively small difference in the clearance of solutes between the different strengths of filters,^33^ as well as the small sample size of the study. The difference in intradialytic unbound cefazolin CL is likely contributed by the dialysis prescription and the different efficiencies of the high-flux dialysis used, which represents 20 years of advancement in the development of dialysis membrane technologies and highlights the ongoing need for pharmacokinetics studies in an advancing field.

We found that weight did not significantly improve the pharmacokinetic model and we report *V* values that are slightly lower than reported in patients with complicated skin-and-soft-tissue infection but without renal impairment (*V* = 25.02 versus 45.2 L, respectively), which may be a consequence of the *V* reported in other studies being based on total concentrations compared with unbound concentrations used in our study.^34^ Furthermore, we observed a higher cefazolin unbound fraction in our population when compared with healthy volunteers (0.38 ± 0.12 versus 0.21).^35,36^ In the absence of hypoalbuminaemia (<24 g/L) in our patient population, the higher unbound fraction can be potentially contributed by the competitive binding of the presence of excess urea (median 19.6 mmol/L).^37,38^ Uraemia has previously been described to decrease protein binding of cefazolin and in turn alter the dialysis removal and likelihood of toxicity.^37,38^ The decreased protein binding may also be explained by the routine use of heparin during dialysis, which has been noted to elevate free fatty acids, which competitively bind to albumin and reduce protein binding of medications.^39^ This is supported by the higher unbound fraction observed in the samples immediately collected before dialysis when compared with those collected immediately after dialysis (mean 36.5% ± 6.8% versus 24.5% ± 14.3%). Higher unbound concentrations equate to increased tissue penetration, which is particularly beneficial for deep-seated infections.

In this study, we have simulated post-dialysis dosing regimens, which eliminates the need for both peripherally inserted central catheters and peripheral venous cannulation, which validates the three-times-weekly post-dialysis regimen being used in some dialysis centres. In this population who are considerably younger than their non-Indigenous counterparts, vein preservation is of high importance as they are likely to require multiple vascular access sites throughout their life.^40^

We have identified some limitations in our study. Firstly, whilst we sampled both unbound and total cefazolin concentrations in the blood, we did not measure the concentrations in tissues, which are more commonly the source of pathogens. Secondly, creatinine concentrations in patients on maintenance dialysis are influenced by IHD and an accurate calculation of residual renal function was not possible. However, we have incorporated TOH in the final pharmacokinetic model to address this limitation. Finally, samples were stored frozen and were required to thaw prior to measurement; though the utmost care was taken to ensure the drug–protein binding re-equilibrated, this may have impacted our measurements of unbound concentrations.

In conclusion, we present a 2 g three-times-weekly post-dialysis dosing regimen for cefazolin, which we recommend when treating bacterial infections with an MIC of ≤2 mg/L. This dosing regimen resulted in no adverse effects nor signs of toxicity. Three-times-weekly post-dialysis dosing reduces the need for both peripherally inserted central catheters and peripheral venous cannulation and spares venous access that may be life-preserving to the patient in years to come.

## Supplementary Material

dkae318_Supplementary_Data

## Supplementary data

Figure S1 and Tables S1 to S4 are available as Supplementary data at *JAC* Online.
