# Supplementary material for: Population pharmacokinetics of unbound cefazolin in infected hospitalized patients requiring intermittent high-flux haemodialysis: can a three-times-weekly post-dialysis dosing regimen provide optimal treatment?
Source: J Antimicrob Chemother. 2024 Sep 10;79(11):2980–9. doi: 10.1093/jac/dkae318 (PMC11531813; doi:10.1093/jac/dkae318)
Supplement: dkae318_Supplementary_Data [file dkae318_supplementary_data.docx]

**Supplementary information**

**Table S1. Cefazolin assay methodology**

| Summary of assay methodology  The total and unbound concentrations of cefazolin in plasma were measured from 1 to 500 mg/L by a validated UHPLC-MS/MS method on a Shimadzu Nexera2 UHPLC system coupled to a Shimadzu 8030+ triple quadrupole mass spectrometer. Test samples were assayed in batches alongside calibrators and quality controls and results were subject to batch acceptance criteria. |
| --- |
| Sample Preparation  The free fraction was first isolated by ultrafiltration at 37°C with Centrifree devices (Merck Millipore, Tullagreen, Ireland), and the ultrafiltered plasma then processed as a typical plasma sample in order to obtain the unbound concentration. To do this, 10 µL of plasma (for total cefazolin) or ultrafiltered plasma (for unbound cefazolin) was combined with stable isotope labelled cefazolin ([^13^C₂,^15^N_1_]-cefazolin, TRC, Toronto, Canada) and then acetonitrile to precipitate proteins. Following centrifugation, the upper layer was isolated for chromatographic analysis. |
| Chromatography  The stationary phase was a Kinetex C8, 1.7 mm, 2.1 x 50 mm analytical column (Phenomenex, Torrence, USA). Mobile phase A was formic acid in water (0.1% v/v) and mobile phase B was formic acid in acetonitrile (0.1% v/v). Mobile phase was delivered as a gradient going from 5% B to 65% B and back again for a 5.0 min run-time. The flow rate was 0.4 mL/min and produced a back pressure of about 3500 psi. |
| Detection  Cefazolin was monitored by positive mode electrospray at MRM of m/z 455.1→323.1. Labelled cefazolin ([^13^C₂,^15^N_1_]-cefazolin) was monitored in positive mode at 458.05→326.05. |
| Validation  The assay method was validated for precision and accuracy using the FDA criteria for bioanalysis. Precision was 5.3, 4.7 and 4.1% and accuracy 0.0, 2.5 and -2.7% at total concentrations of 20, 100 and 400 µg/mL. Unbound concentration precision was 5.4, 3.6 and 6.3%, and fraction unbound was 15.0, 19.1 and 54.6% at total levels of 20, 100 and 400 µg/mL. |

**Table S2. Pharmacokinetic model comparative log-likelihood in (2*LL), AIC and BIC**

| **Model description** | **2*LL** | **AIC** | **BIC** |
| --- | --- | --- | --- |
| **Model development** | | | |
| 1 compartment simple binding | 2410 | 2422 | 2443 |
| 1 compartment complex binding | 2669 | 2681 | 2702 |
| 2 compartment complex binding | 1692 | 1708 | 1736 |
| 2 compartment complex binding with TOH | 1654 | 1670 | 1697 |
| **Backward exclusion** | | | |
| 2 compartment complex binding with TOH | 1654 | 1670 | 1697 |
| 2 compartment complex binding | 1688 | 1705 | 1732 |
| 1 compartment complex binding | 2493 | 2505 | 2525 |
| 1 compartment simple binding | 1903 | 1920 | 1947 |

Abbreviation: TOH, time on haemodialysis; 2*LL, log-likelihood ratio; AIC, Akaike information criterion; BIC, Bayesian information criterion.

| **Table S3.** Pmetrics model file for the final covariate complex binding model |
| --- |
| #Primary variables |
| CL_nHD_, 0.1, 0.8 |
| CL_HD_, 1, 25 |
| V_c_, 3, 9 |
| K_on_, 1, 3 |
| K_off_, 40, 120 |
| K_cp_, 0.1, 13 |
| K_pc_, 0.1, 5.8 |
| #Covariates |
| HDx |
| Alb  RKF |
| #Secondary variables |
| B_max1_=Alb*V_c_*4.1 |
| CL=CL_nHD_*(59/TOH)**0.28 |
| &IF (HDx.EQ.1) CL=CL_HD_ |
| K_e_=CL/V_c_ |
| #Differential equations |
| XP(1) = RATEIV(1) - (K_e_+K_cp_)*X(1) - (K_on_/V_c_)*(B_max1_-X(2))*X(1) + K_off_*X(2) + K_pc_*X(3) |
| XP(2) = (K_on_/V_c_)*(B_max1_-X(2))*X(1) - K_off_*X(2) |
| XP(3) = K_cp_*X(1) - K_pc_*X(3) |
| #Output equations |
| Y(1) = X(1)/V_c_ |
| Y(2) = (X(2) + X(1))/V_c_ |
| #Error |
| G=2 |
| 0.3,0.1,0,0 |
| 0.3,0.1,0,0 |
| CL_nHD_, CL when patient is not on dialysis (L.h^-1^); CL_HD_, CL during dialysis; V_c_, V in central compartment (L); K_on_, second-order association rate constant (L.mg^-1^.h^-1^); K_off_, first-order dissociation rate constant (h^-1^); K_cp_, first-order rate constant for distribution from central to peripheral compartment (h^-1^); K_pc_, first-order rate constant for distribution from peripheral to central compartment (h^-1^); HDx, haemodialysis; Alb, serum albumin concentration (g/L); TOH, time patient has been on haemodialysis; B_max1_, maximum binding mass of cefazolin (mg); K_e_, first-order elimination rate constant (h^-1^); XP(n), notation for dX(n)/dt where n is the compartment number; RATEIV(1), notation to indicate an infusion of drug (1); X(n), amount of drug in compartment where n is the compartment number; Y(1), concentration of unbound drug in the central compartment; Y(2), concentration of total drug in the central compartment; IF (HDx.EQ.1) CL= CL_nHD_, when haemodialysis is on, CL is represented by CL_HD_. |

**Table S4. Dialysis parameters**

| **Dialysis parameters** |  |
| --- | --- |
| Number of patients on haemodialysis | 16 |
| Number of patients on haemodiafiltration | 0 |
| Blood flow rate, mL/min | 348 ± 47 |
| Ultrafiltration volume, mL | å2939 ± 819 |
| Kt/V | 1.69 ± 0.37 |
| Recirculation, % | 11.4 ± 1.9 |

Abbreviation: K, dialysis clearance of urea; t, dialysis time; V, volume of distribution of urea.

Data presented in mean (SD).

**Figure S1. Diagram to show the compartments of the final pharmacokinetic model**


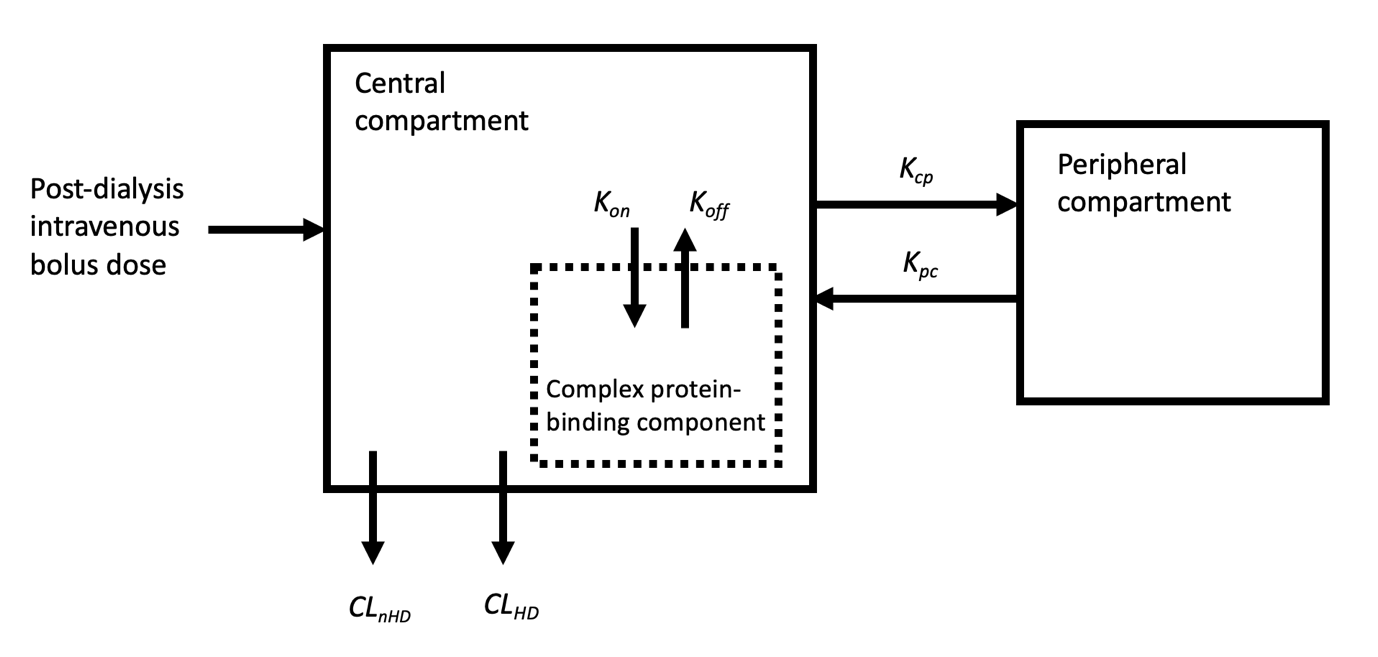


Abbreviation: K_on_, second-order association rate constant; K_off_, first-order dissociation rate constant; K_cp_, first-order rate constant for distribution from central to peripheral compartment; K_pc_, first-order rate constant for distribution from peripheral to central compartment; CL_nHD_, drug clearance when patient is not on dialysis; CL_HD_, drug clearance during dialysis
